# Exploring the Acceptability and Suitability of Synchronous Online Focus Groups for Health Research With Métis Nation of Ontario Citizens: An Internet-Based Survey

**DOI:** 10.2196/70986

**Published:** 2025-10-09

**Authors:** Abigail J Simms, Noel Tsui, Robynn Sadler, Amy Mersereau, Cindi Rye, C David Crenna, Jeff Evenson, Sarah A Edwards

**Affiliations:** 1 Housing and Infrastructure Branch, Métis Nation of Ontario, Ottawa, ON, Canada; 2 Data and Analytic Services, ICES (formerly the Institute for Clinical Evaluative Sciences), V1 06, 2075 Bayview Avenue, Toronto, ON, M4N 3N5, Canada; 3 Dalla Lana School of Public Health, University of Toronto, Toronto, ON, Canada; 4 Priority Decision Data, Perth, ON, Canada; 5 Edgewood Associates, Toronto, ON, Canada

**Keywords:** Indigenous health, Métis health, acceptability, virtual research methods, synchronous virtual focus group

## Abstract

**Background:**

The COVID-19 pandemic forced many researchers to adjust research methods from in-person to online formats. This paper explores the acceptability and suitability of synchronous online focus groups used to explore housing and health with Métis Nation of Ontario (MNO) citizens, one of 3 constitutionally recognized Indigenous Peoples in Canada.

**Objective:**

The objective of this internet-based survey was to understand the experiences of MNO citizens participating in synchronous online focus groups.

**Methods:**

Only participants of the “Understanding Housing and Health” project were eligible to complete the survey and were recruited via a ‘thank you’ email. The survey asked respondents to rate their experience, satisfaction, and preference, as well as the feasibility and cultural appropriateness of the online focus group. An open textbox allowed respondents to share additional thoughts. Demographic and personal information (ie, age, gender, MNO region, and email) were collected. A total of 33/35 eligible participants completed the survey. A content analysis was conducted to generate themes from the open textbox responses and used to triangulate the results. The survey was developed collaboratively with MNO staff.

**Results:**

Most respondents identified as women and were 45‐65 years and older. All respondents had used Zoom before, and most (n=28, 85%) were either strongly or somewhat confident in their ability to use Zoom. One hundred percent of respondents strongly or somewhat agreed that they would participate in an online focus group in the future, and 86% of respondents strongly (n=22, 67%) or somewhat (n=7, 21%) agreed that an online focus group was culturally appropriate for Métis health research. A total of 82% (n=27) of respondents strongly or somewhat agreed that an online focus group was more feasible. Moreover, 58% (n=19) of respondents strongly or somewhat disagreed that they would have preferred to participate in an in-person focus group, while 27% (n=9) were neutral. Around 58% (n=19) of respondents could see other participants all of the time and did not experience lag at any point, while 25 (76%) could hear other participants all of the time, indicating fewer issues with audio. A total of 70% (n=23) of respondents felt they could connect with others all or most of the time, while 30% (n=10) felt they could do this some of the time or rarely. Content analysis of the open textbox responses generated 4 themes: internet and technology issues, accessibility, structure of the group discussion, and positive feedback.

**Conclusions:**

The use of online focus groups for research with MNO citizens is acceptable; however, internet and technology issues can affect a participant’s ability to fully engage. Considerations around cultural appropriateness and connecting with others should be made. This information will help inform method selection for future research work conducted in collaboration with the MNO.

## Introduction

The COVID-19 pandemic forced a pivot to online platforms for face-to-face research activities such as focus groups, which impacted research projects conducted by the Métis Nation of Ontario (MNO) [1-3]. The MNO represents the rights and interests of Métis in Ontario, Canada, and provides many social, justice, and education programs for its citizens and conducts relevant research on their behalf [4]. The MNO registry comprises more than 30,000 citizens and recently underwent a comprehensive review [4,5]. Using the national definition of Métis, the MNO registry determines citizenship for an applicant based on (1) self-identifying as Métis, (2) being of historic Métis Nation ancestry, (3) being distinct from other Indigenous Nations, (4) being accepted by the Métis Nation [5]. The Métis are one of 3 recognized Indigenous Nations with the section 35 rights under Canada’s constitution. The Métis are descendants of First Nations and European settler ancestry that formed distinct communities via ethnogenesis along the waterways and lakes of Ontario, as well as the 3 prairie provinces, parts of British Columbia, the Northwest Territories, and Northern United States [6]. Métis have a distinct history, language, and culture, though there are regional differences [6]. The MNO pivoted to adapt to the COVID-19 pandemic. Online communication tools, such as Facebook (Meta), Instagram (Meta), X (formerly Twitter), the MNO website, and email list services, made up an important part of MNO communications with citizens throughout the COVID-19 pandemic. Online town halls were also used to connect with MNO citizens to share pandemic updates and to host question-and-answer sessions with Métis doctors on the SARS-CoV-2 virus and vaccines [7]. Other major community events also shifted online, such as the MNO’s Annual General Assembly.

The need to rely on Zoom (Zoom Video Communications, Inc) to interact with Métis citizens for research purposes during the COVID-19 pandemic posed important questions around the acceptability and suitability of online data collection for this population since visiting with one another and spending time face-to-face is an important part of traditional ways of knowing and being for Métis people [8]. Visiting in a Métis context has even been adapted as a health research approach [9]. Interest in what may be considered a culturally appropriate data collection method for research with Indigenous Peoples led to a review of the literature conducted by Wright et al [10]. They identified photovoice, symbol-based reflection, circles, and story-telling oral-data collection methods as being rigorous and culturally appropriate for research with Indigenous Peoples in Canada [10]. This review specifically excluded interviews and focus groups due to concerns that they may not always be culturally appropriate for this population [10,11]. However, a systematic review by Hunt and Young [12], looking at studies using group-based qualitative methods for health research with Indigenous children, found that many studies incorporated aspects of Indigenous sharing circles into Western focus groups to produce a hybrid method that was culturally appropriate and safe for research with this population, which is in alignment with our focus group method. Globally, many other researchers have engaged focus groups, both online and in-person, in community-based participatory study designs with Indigenous Peoples exploring topics such as consent in research, housing, dementia, and traditional medicines, among others [13-17].

Although we were forced to use online tools over more traditional in-person, face-to-face ways of collecting information, online data collection takes advantage of technological advancements and offers a variety of benefits [2,3,18-21]. Our survey asked MNO citizen participants of online synchronous focus groups about their experience with the online data collection method, focusing on acceptability and suitability. Synchronous focus groups involve participants meeting on an online platform (ie, Zoom, Skype; Microsoft, etc) and having a facilitated discussion in real-time [22]. Online delivery for focus groups reduces costs for researchers since transportation and renting space are no longer required. The logistics of arranging meetings with several groups of participants are also simplified since it is easier to set up meetings online than in-person, which speeds up data collection and reduces participant burden [23-26]. There is also more flexibility around meeting times and around canceling and rescheduling for online delivery [18]. Participants living great distances apart can be brought together with the use of an online platform which may allow for the collection of more diverse information [27]. In addition, researcher bias can be reduced, and participants may feel less pressure to conform to other opinions expressed in the group since they are not in the same physical space [23,28]. Some richness of information may be lost, however, due to body language being harder to communicate and capture over online platforms [22].

More academic literature was produced on the use of online data collection methods during COVID-19. In 2 papers reflecting on online focus groups, it was determined that this method is feasible for different populations and that sufficient interaction can be achieved with a skilled moderator [29,30]. Coming out of the COVID-19 pandemic, Indigenous researchers are reimagining Indigenous data collection approaches for online spaces, such as Cooms et al’s [31] exploration of online yarning using Facebook, an Indigenist research method belonging to First Nations in Australia. This survey adds more to the literature concerning the use of synchronous online focus groups from the perspective of Métis people, one that had yet to be explored.

## Methods

### Overview

This voluntary internet-based survey used a convenience sample and recruited respondents via a ‘thank you’ email sent to participants at the end of synchronous online focus groups conducted for the MNO “Understanding Housing and Health” project between August 2022 and February 2023.

### Summary of the “Understanding Housing and Health Project”

The “Understanding Housing and Health” project was meant to better understand the relationship between housing and health for MNO citizens and to provide important feedback to improve the MNO’s existing housing programs [32]. A purposive sample of participants was recruited using the MNO’s social media channels and website. Eligibility criteria included being (1) a registered MNO citizen, (2) a current resident within Ontario, and (3) 18 years of age or older. A total of 7 online focus groups were conducted, with 3‐9 participants per group and 35 participants overall. The average time for the focus groups was not reported. All focus groups were recorded using the Zoom record feature, with consent from participants, and transcript summaries were shared back for participant verification. Participants were emailed the questions and Zoom 101 instructions before their scheduled online focus group. Before starting the focus groups, an MNO Senator would share a prayer to start the group in a good way, and both AJS and NT would introduce themselves and the research, including the purpose and goals. The MNO Senator would also close the group with a prayer and offer their reflections on the discussion. The focus group guide was created and pilot tested within the research team.

### Eligibility Criteria

Only participants who had completed an online focus group for the aforementioned study were eligible to complete this survey via Qualtrics (Qualtrics International Inc) [33].

### Survey Design and Structure

Respondents were asked 20 questions total (15 Likert, 1 open textbox response, and 4 demographic and personal information). The Likert style questions were either 3- or 5-point and asked respondents to rate their experience, satisfaction, preference, feasibility, and the cultural appropriateness of the online focus groups using the Zoom Video Conference tool. An open textbox was included for respondents to share any other thoughts they had about their online focus group experience. The survey was planned to take 5‐10 minutes to complete. Around 1-5 questions were displayed per page over 7 pages (including a thank you message at the end). All questions were forced responses, except for the open textbox and nonresponse options were not provided. Adaptive questioning was not needed. The survey questions were based on a satisfaction survey distributed to Black sexual minority men after completing an online synchronous focus group on HIV prevention conducted by Dangerfield II et al [34] with additions made by MNO staff to account for cultural relevance for Métis citizens. Demographic and personal information (age, gender, MNO Region, and email) was also collected. The MNO has 9 regions that divide up the province of Ontario and represent administrative borders. It is the MNO’s preference that the location be reported by the MNO Region. A copy of the survey is available in the Multimedia Appendix 1. The CHERRIES (Checklist for Reporting Results of Internet E-Surveys) checklist was used to report the findings of this survey, and the study design domain of COREQ (Consolidated Criteria for Reporting Qualitative Research) checklist was used to summarize select information from the qualitative “Understanding Housing and Health” project [35,36].

### Métis Nation of Ontario and ICES Data Sharing Agreement

This survey was created through collaboration between the MNO and ICES (formerly the Institute for Clinical and Evaluative Sciences) seconded staff. This research relationship works to advance the data sovereignty of MNO citizens and produces important information about citizens’ health and wellness needs for the MNO [37]. As part of this agreement, 2 epidemiologists (AJS and NT) and a staff scientist (SAE) are seconded from ICES to the MNO and work exclusively on Métis health and wellness research at the direction of the MNO. The data from this project is not held at ICES and is kept on MNO secure servers. As the representative body of Métis in Ontario, the MNO conducts research on behalf of its citizens with the purpose of closing health and wellness knowledge and outcome gaps.

### Data Analysis

Survey data were analyzed using descriptive statistics. An inductive thematic analysis of the open textbox responses was conducted by coders AJS and NT using Microsoft Excel. Both coders independently coded the responses and discussed the themes they observed. These themes were defined, and a codebook was created that was applied to all responses. There was a total of 14 open textbox responses. Three nonspecific answers, “no, all good,” “nothing,” and “N/A,” were not included in the analysis, giving a total of 11 open textbox responses for the content analysis.

### Reflexivity

The research team for the bigger “Understanding Housing and Health” project was comprised of Master of Public Health students, experienced PhD public health researchers, topic area specialists, community members, MNO staff, and MNO Senators. AJS and NT, who both identify as women, were Master of Public Health students during this project and took turns facilitating or taking notes for the focus groups. Before conducting the online focus groups, they closely reviewed the literature and sat in on a focus group to gain observational experience. AJS is also Métis and an MNO citizen.

### Ethical Considerations

This survey was approved by the MNO and the University of Toronto Health Sciences Research Ethics Board (RIS Human Protocol Number 42320). One informed consent form was read and signed by all participants for both the online focus groups and this survey. This was done before completing a screening survey for the “Understanding Housing and Health” project. The informed consent form did not describe the length of the survey, but this information was shared during the online focus groups. The form also states that only deidentified information would be used for analysis and stored on a secure and confidential server. The primary investigator was identified, and a contact phone number was provided. Both the screening survey and this internet-based survey were delivered using Qualtrics survey software (Qualtrics International Inc) [33]. It was made clear in this consent process that respondents could stop participating at any time for any reason without consequence. Respondents who completed the survey received a CA$5 (US$3.49) gift card for their time.

## Results

### Participant Characteristics

**Figure 1.**
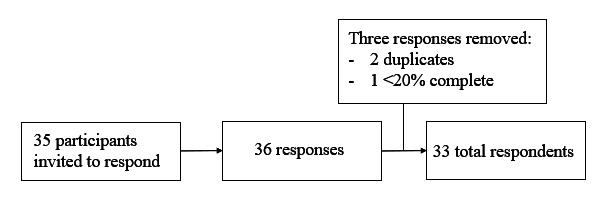
Flow chart depicting the number of participants invited to complete the voluntary internet-based survey about their online focus group experience, the initial number of responses, the number of excluded responses, and the total number of responses.

Thirty-three of 35 eligible respondents completed the survey (94% completion rate) and 34 of 35 started the survey (97% participation rate). Only results from unique visitors (unique name and IP address) were included in the analysis. Three responses were deleted from the dataset because they were either multiple entries (2) or due to the lack of completeness (>20%; 1) (Figure 1). We did not collect or receive reasons why participants chose not to complete the survey.

Most respondents (n=24, 75%) identified as cis-women, and 58% (n=19) were 45‐65 years and older. Additional gender identities included in the survey included trans woman, trans man, genderqueer, genderfluid, androgynous, and nonbinary, but no respondents selected these options. Twenty-one percent (n=7) of respondents selected “identity not listed” and provided a response in an open textbox. One open textbox response was left blank, while other respondents answered either “woman,” “man,” or “female”. These responses were added to the “cis-woman” or “cis-man” counts and indicate that the terms “cis-woman” and “cis-man” were not readily understood by respondents. There are 9 MNO Regions in Ontario. For the MNO Region, 21% (n=7) of respondents indicated they were from Region 7, and 18% (n=6) indicated they were from Region 6. Both regions are in Southern Ontario and Region 7 has the highest population of MNO citizens. A detailed breakdown of MNO Regions is not provided in Table 1 since fewer than 6 participants selected any of the other MNO Regions. Counts less than 5 are either not reportable or are suppressed to minimize the risk of reidentification. This is also why “cis-man” and Two-Spirit are combined in Table 1.

**Table 1.** This table provides demographic information on the self-reported age and gender by Métis online focus group respondents for our acceptability and suitability survey on the use of online focus groups for this project. Participant Characteristics.

Participant characteristics	Count (N=33), n (%)	
Age (years)		
18‐24	0 (0)	
25‐44	14 (42)	
45‐65+	19 (58)	
Gender^a^		
Cis-woman	24 (75)	
Cis-man and Two-Spirit	8 (25)	

a One participant did not indicate gender

### Participant Zoom Video Conference Tool Experience

All respondents had prior Zoom experience, and 70% (n=23) of respondents indicated that they used ‘Zoom regularly for work, school, personal use, etc’ (see Table 2). The majority (n=28, 85%) were also strongly confident 64% (n=21) or somewhat confident 21% (n=7) in their ability to use Zoom.

**Table 2.** This table summarizes responses to our survey questions exploring respondents’ Zoom conference platform experience and confidence.

Question	Response option	Participants, n (%)
Please select the statement that best describes your use of the Zoom Video Conference Tool	This was my first time using Zoom I have used Zoom before, but I do not use Zoom regularly I use Zoom regularly for work, school, personal use, etc	0 (0) 10 (30) 23 (70)
I am confident in my ability to use the Zoom Video Conference tool	Strongly agree Somewhat agree Neither agree nor disagree Somewhat disagree Strongly disagree	21 (64) 7 (21) 1 (3) 3 (9) 1 (3)

### Acceptability of Focus Groups

Seventy-six percent (n=25) of respondents strongly agreed that they were satisfied with participating in an online focus group (see Table 3). Fifty-two percent (n=17) strongly disagreed that they would have preferred to participate in an in-person focus group, although 27% (n=9) of respondents were neutral for this statement, and 15% (n=5) would have preferred an in-person focus group. All respondents strongly agreed 88% (n=29) or somewhat agreed 12% (n=4) that they would want to participate in an online focus group in the future. Seventy-three percent (n=24) of respondents strongly agreed that it was more feasible for them to participate in an online focus group than an in-person focus group. The majority of respondents strongly (n=22, 67%) or somewhat (n=7, 21%) agreed that it was culturally appropriate to conduct online focus groups with MNO citizens.

Eighty-eight percent (n=29) of respondents strongly agreed or somewhat agreed that they believed the research team would keep their information confidential, while 76% (n=25) of respondents strongly or somewhat agreed that other participants would keep their information confidential (see Table 3). Almost all 94% (n=31) of respondents strongly agreed and somewhat agreed that they believed other participants were in a private space for the online focus groups. Finally, just over three-quarters of respondents strongly agreed or somewhat agreed that they were satisfied with their compensation for participating in an online focus group.

**Table 3.** This table summarizes responses to our survey questions exploring participant satisfaction with the online focus group method, feasibility, cultural appropriateness, compensation, and participant privacy.

Question, total (N=33)	Response option, n (%)				
	Strongly agree	Somewhat agree	Neither agree nor disagree	Somewhat disagree	Strongly disagree
I was satisfied with participating in a focus group online	25 (76)	6 (18)	1 (3)	0 (0)	1 (3)
I would have preferred to participate in the focus group in-person	0 (0)	5 (15)	9 (27)	2 (6)	17 (52)
In the future, I would like to participate in other online focus groups	29 (88)	4 (12)	0 (0)	0 (0)	0 (0)
It is more feasible for me to participate in an online focus group than in an in-person focus group	24 (73)	3 (9)	6 (18)	0 (0)	0 (0)
I believe it is culturally appropriate to use online focus groups for research involving citizens of the Métis Nation of Ontario	22 (67)	7 (21)	2 (6)	1 (3)	1 (3)
I felt that my information will be kept confidential by the research team	24 (73)	5 (15)	3 (9)	0 (0)	1 (3)
I believe other participants were in a private space	21 (64)	10 (30)	1 (3)	1 (3)	0 (0)
I believe my information will be kept confidential by the other people in the focus group	18 (55)	7 (21)	6 (18)	2 (6)	0 (0)
I was satisfied with the compensation I received for participating in the study	22 (67)	4 (12)	5 (15)	1 (3)	1 (3)

### Suitability of Online Focus Groups using the Zoom Video Conference Tool

Only 58% (n=19) of respondents could see other participants all of the time (video quality). However, 76% (n=25) of respondents could hear other participants all of the time (audio quality), and 42% (n=14) did experience some lag in video or audio quality. Finally, just over half of the respondents felt they could connect with other participants during the focus group, while the other half felt less confident that they were connecting with others (see Table 4).

**Table 4.** This table summarizes respondents’ technology and internet experiences (ie, audio, video, and lag connecting with other participants) to explore the suitability of the online focus group method.

Question, total N=33)	Response option, n (%)				
	All of the time	Most of the time	Some of the time	Rarely	None of the time
I was able to see other participants during the focus group discussion (video quality)	19 (58)	10 (30)	4 (12)	0 (0)	0 (0)
I was able to hear other participants during the focus group discussion (audio quality)	25 (76)	6 (18)	2 (6)	0 (0)	0 (0)
I was able to hear and see other participants without delays in the live feed (lag in audio and video)	19 (58)	11 (33)	3 (9)	0 (0)	0 (0)
I feel that I am able to connect with other participants in a meaningful way during the focus group discussion	17 (52)	6 (18)	9 (27)	1 (3)	0 (0)

### Open Textbox Responses

There were 11 open textbox responses analyzed. Three themes were generated from this analysis: *Internet and technology issues*, *Accessibility*, *Structure of the group discussion*, and *Positive feedback. Internet and technology issues* included audio distortion, and losing connectivity or having poor connectivity. One respondent shared, “Zoom works best when people agree to be on camera and on audio. If people are just typing and few on video it doesn’t make for great discussion.”

As facilitators, we chose to allow participants to contribute their ideas in the Zoom chat, particularly if they were having internet or technology issues that prevented video and audio, as this respondent did, “I had to use the chat to communicate because of poor connection on my end. It worked.” The previous quote is an example of the *Structure of the group discussion,* which included keeping participants more on topic, having a different opening that doesn’t include the Lord’s prayer, and enjoying the small group sizes and mixed MNO Regions. Having mixed MNO Regions reduced the likelihood of participants knowing each other based on living in the same MNO Region. *Accessibility* included a request to ensure closed captions are turned on and to supply some technology equipment to support joining a Zoom call. One respondent also shared that joining the focus group virtually is “…easier for me than in-person.” A couple *Positive feedback* comments were shared like “great group of people.”

## Discussion

### Principal Findings

To our knowledge, this is the first study to explore the acceptability and suitability of an online focus group data collection method for a Métis health and wellness study. Overall, most aspects of the online focus groups were rated highly for acceptability, with the lowest-rated aspects being around privacy, cultural appropriateness, and ability to connect with others. The only other study to explore methods acceptability with Métis research participants was for blood spot testing for HIV [38]. With the advancement of technology for both the medical field and for health and wellness research, it is important to understand what is and is not acceptable or suitable for Métis people. This question is particularly important in the MNO context, since Ontario is a large province with MNO citizens living in urban, rural, and remote regions, which produces costly and time-consuming barriers for research that all citizens have a right to participate in if they are eligible. Online ways to connect with MNO citizens for research purposes offer the potential to reduce the time, cost, and travel burden for both participants and researchers. This is an important consideration for projects with populations that are similarly spread out, have financial and time restrictions, or that are working with populations that have barriers to transportation [39].

During the COVID-19 pandemic, other groups conducting research with Indigenous Peoples shifted to online data collection methods with more consideration being given to translating Indigenist research approaches to online spaces [14,31]. Moreover, the use of online spaces and technology for data collection may help to better include historically excluded groups from research, like the Métis, as Folk et al [40] discuss in their reflection on using online focus groups to explore physical activity with Black and African American women and children. Similar to our open textbox themes, this group’s main lessons learned included participant privacy, technological challenges and time burdens, and participant engagement. Other important lessons they shared include the importance of community-building, flexibility, and understanding social context when conducting online focus groups [40]. Similarly, Lathen and Laestadius [41] shared important insights comparing in-person versus online focus groups conducted during the COVID-19 pandemic from their work with low socio-economic status African American adults. Many of the benefits we see as researchers for using online spaces for focus groups can create barriers for some participants, such as access to technology, incurring data costs to participate, and needing to spend money on transport to get to a computer or public Wi-Fi, among others. These researchers also reported that everyone in their online focus group (participants, facilitators, and assistants) had a more difficult time building rapport and connecting with others in the group, which is consistent with our survey results [41].

One hundred percent of respondents strongly or somewhat agreed that they would participate in an online focus group in the future, and most respondents strongly (n=22, 67%) or somewhat (n=7, 21%) agreed that an online focus group was culturally appropriate for Métis health research; however, fewer respondents strongly agreed with the latter statement in comparison to other statements of which most were strongly agreed upon. In addition, 82% (n=27) of respondents strongly or somewhat agreed that an online focus group was more feasible for them; however, results were more mixed for the statement asking respondents if they would have preferred to participate in an in-person focus group, with 52% (n=17) strongly disagreeing, 27% (n=9) being neutral, and 15% (n=5) somewhat agreeing. These results contrast somewhat with those found by Dangerfield II et al’s [34] work assessing the acceptability of online synchronous focus groups among Black sexual minority men. In their work, all participants agreed or strongly agreed that they were satisfied with an online focus group, while only 94% (n=31) of our respondents were strongly or somewhat satisfied [34]. Moreover, 100% of their participants agreed or strongly agreed that their information would be kept safe by others in their group [34]. For our results, only 76% (n=25) (strongly and somewhat agreed) of participants felt their information would be kept confidential by the group, while 18% (n=6) were neutral, and 6% (n=2) disagreed.

The findings from our open textbox responses may give insight into why respondents felt this way about confidentiality. One respondent replied that they appreciated that the groups were made up of participants from different MNO Regions, so that it was less likely someone would know you. As part of the introduction to the focus group, we included ground rules that asked participants to respect the privacy and confidentiality of others in the group by refraining from repeating information outside of the group. However, we cannot control if this is adhered to, which may result in some distrust between participants in our focus groups and may affect confidence around privacy. Some participants were also having difficulty with audio and video and may have missed some of these instructions, which could lead them to believe they were not covered in enough detail. In the future, these ground rules will be emailed to participants with the questions we send ahead of time, in addition to being covered verbally before the group starts.

For suitability, only 58% (n=19) of participants could see other participants all of the time and did not experience lag at any point, while 76% (n=25) could hear other participants all of the time. Fifty-two percent (n=17) of respondents felt they were able to connect with other participants in a meaningful way. Some respondents elaborated on their technology issues in their open textbox responses. Due to connectivity issues, some participants had no choice but to keep their cameras off and use the chat function to participate during the online focus group. But as 1 participant responded, they felt it would have been a better experience if all participants had their cameras on and contributed to the discussion orally, which they felt would allow for more connection. As such, online focus groups may not be for everyone. Technology issues could be addressed by sending participants headphones ahead of time or arranging for them to use a computer at the nearest MNO office. Internet connectivity, especially in rural and remote areas of Ontario, is more difficult to address.

Similar to other studies, we found that online focus groups were very convenient to implement, since renting space and organizing transportation was replaced by a Zoom link and rescheduling was considerably easier [2,3,42]. The use of online focus groups for research with MNO citizens is acceptable; however, internet and technology issues can affect a participant’s ability to fully engage. Considerations around the limitations of traditional ways of sharing and gathering knowledge for the Métis people in online spaces and the ability to create connections with and between participants should also be considered [41].

### Limitations

This study has a few limitations. Self-selection bias of participants needs to be considered. Participants who are more accepting of online data collection methods may have been more likely to self-select into a study using them, influencing our survey results. Second, the study topic could also influence the acceptability of using an online data collection method. For example, some research topics can be triggering, such as those that involve discussions related to mental health, and it is more difficult to provide crisis intervention over an online platform, which may make certain conversations more appropriate in-person. Third, this research was conducted during the COVID-19 pandemic when it was not safe to gather in groups and was a driving factor for us choosing an online method in the first place. In Ontario, Canada, COVID-19 continues to circulate, although there are no longer any restrictions on gathering [43]. Participants’ responses may have differed if the online focus groups were conducted during a stricter period of the COVID-19 pandemic. However, our research group continues to use online platforms for MNO health and wellness research, and citizens continue to participate. Finally, this survey explored participants’ experiences at a high level and did not include an open textbox response for each question, which may have resulted in more detailed insights. In the future, if we select to use online focus groups, we may include time at the end to allow participants to give more detailed feedback about their experience with the method. Although there was repetitiveness in the open textbox responses that aligned with results from our other questions, it is unlikely that data saturation was reached due to the limited number and sparseness of the open textbox responses. Including an acceptability and feasibility survey for other projects that use online group methods will help us better tailor online approaches for research with MNO citizens.

### Conclusions

This paper contributes to the growing literature on the use of online focus groups as a method to collect information and stories for health research. Our survey specifically explored the use of this method with the Métis people (MNO citizens) and concludes that it may be acceptable, but important considerations around technology and internet barriers, the ability of the group to connect with each other, and traditional knowledge sharing for the Métis people should be considered. This information is valuable for guiding the selection of methods for future research with the Métis and for other populations facing similar barriers.

## Supplementary material

10.2196/70986 Multimedia Appendix 1 Acceptability and suitability survey questions on the use of online focus groups for the qualitative “Understanding Housing and Health” project.

## Abbreviations

CHERRIES: Checklist for Reporting Results of Internet E-Surveys
COREQ: Consolidated Criteria for Reporting Qualitative Research
ICES: Institute for Clinical and Evaluative Sciences
MNO: Métis Nation of Ontario

## References

[1] Simms AJ, King KD, Tsui N, Edwards SA, Mecredy G, Métis Nation of Ontario (2023). COVID-19 vaccine behaviour among citizens of the Métis Nation of Ontario: a qualitative study. Vaccine (Auckl).

[2] Dodds S, Hess AC (2021). Adapting research methodology during COVID-19: lessons for transformative service research. JOSM.

[3] Eigege CY, Daundasekara SS, Gomez ML, Walton QL, Hernandez DC (2022). Conducting research six feet apart: the feasibility of transitioning qualitative research to meet the emerging research needs during a pandemic. Int J Qual Methods.

[4] The Métis Nation of Ontario.

[5] Métis Nation of Ontario – Fact Sheet: Implementation of the National Definition of Métis within Ontario and the MNO Registry Policy. Métis Nation of Ontario.

[6] Culture & heritage. Métis Nation of Ontario.

[7] (2021). All MNO citizen online town hall. Métis Nation of Ontario.

[8] Macdougall B National Collaborating Centre for Aboriginal Health. Land, family and identity: Contextualizing Métis health and well-being.

[9] Gaudet JC (2019). Keeoukaywin: the visiting way - fostering an Indigenous research methodology. aboriginal policy stud.

[10] Wright A, Wahoush O, Ballantyne M, Gabel C, Jack S (2016). Qualitative health research involving Indigenous Peoples: culturally appropriate data collection methods. TQR.

[11] Smith L (1999). Decolonizing Methodologies: Research and Indigenous Peoples.

[12] Hunt SC, Young NL (2021). Blending Indigenous sharing circle and Western focus group methodologies for the study of Indigenous children’s health: a systematic review. Int J Qual Methods.

[13] Sitotaw R, Lulekal E, Abate D (2020). Ethnomycological study of edible and medicinal mushrooms in Menge District, Asossa Zone, Benshangul Gumuz Region, Ethiopia. J Ethnobiol Ethnomed.

[14] Lambrou NH, Blind MJ, Ketcher D (2022). Indigenous cultural understandings of Alzheimer’s disease and related dementias – research and engagement (ICARE): relationships between cultural strengths, education and stigma. Alzheimers Dement.

[15] Jacklin K, Ly A, Calam B, Green M, Walker L, Crowshoe L (2016). An innovative sequential focus group method for investigating diabetes care experiences with Indigenous Peoples in Canada. Int J Qual Methods.

[16] Agudelo-Hernández F, Coral-Vela LP, Pabuena-Yepes LH (2025). Mental health risk communication and community participation among an Indigenous people in Colombia. Rev Panam Salud Publica.

[17] Andersen MJ, Williamson AB, Fernando P, Redman S, Vincent F (2016). “There’s a housing crisis going on in Sydney for Aboriginal people”: focus group accounts of housing and perceived associations with health. BMC Public Health.

[18] Kite J, Phongsavan P (2017). Insights for conducting real-time focus groups online using a web conferencing service. F1000Res.

[19] Gray LM, Wong-Wylie G, Rempel GR, Cook K (2020). Expanding qualitative research interviewing strategies: Zoom Video Communications. TQR.

[20] Halliday M, Mill D, Johnson J, Lee K (2021). Let’s talk virtual! Online focus group facilitation for the modern researcher. Res Social Adm Pharm.

[21] Menary J, Stetkiewicz S, Nair A (2021). Going virtual: adapting in-person interactive focus groups to the online environment. Emerald Open Research.

[22] Liamputtong P, Liamputtong P (2011). Focus Group Methodology: Principles and Practice.

[23] Gaiser TJ, Feilding N, Lee RM, Blank G (2008). The Sage Handbook of Online Research Methods.

[24] Mann C, Stewart F (2000). Internet Communication and Qualitative Research: A Handbook for Researching Online.

[25] Valaitis RK, Sword WA (2005). Online discussions with pregnant and parenting adolescents: perspectives and possibilities. Health Promot Pract.

[26] Liamputtong P (2006). Health Research in Cyberspace: Methodological, Practical and Personal Issues.

[27] Matthews KL, Baird M, Duchesne G (2018). Using online meeting software to facilitate geographically dispersed focus groups for health workforce research. Qual Health Res.

[28] Reid DJ, Reid FJM (2005). Online focus groups: an in-depth comparison of computer-mediated and conventional focus group discussions. Int J Market Res.

[29] Henage CB, Ferreri SP, Schlusser C (2021). Transitioning focus group research to a videoconferencing environment: a descriptive analysis of interactivity. Pharmacy (Basel).

[30] Cordell A, Wilk C, Orsulic-Jeras S, Powers S, Ejaz F, Sanders L (2021). The focus group must go on: lessons learned from conducting virtual focus groups. Innov Aging.

[31] Cooms S, Leroy-Dyer S, Muurlink O (2025). The rise of virtual yarning: an Indigenist research method. Qual Res.

[32] Tsui N, Simms AJ, Lacka C (2025). Exploring the relationship between housing conditions and Métis Nation of Ontario citizen’s health: a qualitative study. BMC Public Health.

[33] (2023). Qualtrics.

[34] Dangerfield Ii DT, Wylie C, Anderson JN (2021). Conducting virtual, synchronous focus groups among Black sexual minority men: qualitative study. JMIR Public Health Surveill.

[35] Eysenbach G (2004). Improving the quality of Web surveys: the Checklist for Reporting Results of Internet E-Surveys (CHERRIES). J Med Internet Res.

[36] Tong A, Sainsbury P, Craig J (2007). Consolidated criteria for reporting qualitative research (COREQ): a 32-item checklist for interviews and focus groups. Int J Qual Health Care.

[37] ICES and Métis Nation of Ontario partner on new agreement. IC/ES.

[38] Landy R, Atkinson D, Ogilvie K (2022). Assessing the acceptability of dried blood spot testing for HIV and STBBI among Métis people in a community driven pilot project in Alberta, Canada. BMC Health Serv Res.

[39] Mabragaña M, Carballo-Diéguez A, Giguere R (2013). Young women’s experience with using videoconferencing for the assessment of sexual behavior and microbicide use. Telemed J E Health.

[40] Folk AL, Grace SM, Urvig M, Barr-Anderson DJ (2025). Virtual focus groups on Zoom: “Lessons Learned” from two physical activity studies among Black and African American women and children. Int J Soc Res Methodol.

[41] Lathen L, Laestadius L (2021). Reflections on online focus group research with low socio-economic status African American adults during COVID-19. Int J Qual Methods.

[42] Irani E (2019). The use of videoconferencing for qualitative interviewing: opportunities, challenges, and considerations. Clin Nurs Res.

[43] (2022). Post-COVID-19 condition in canada: what we know, what we don’t know, and a framework for action. Government of Canada.

