# Supplementary material for: Exploring the Acceptability and Suitability of Synchronous Online Focus Groups for Health Research With Métis Nation of Ontario Citizens: An Internet-Based Survey
Source: JMIR Form Res. 2025 Oct 9;9:e70986. doi: 10.2196/70986 (PMC12510437; doi:10.2196/70986)
Supplement: Multimedia Appendix 1 [file formative-v9-e70986-s001.docx]

Appendix 1

Table 1: Acceptability and suitability survey questions on the use of virtual focus groups for the qualitative “Understanding Housing and Health” project. These questions were asked via an online survey using Qualtrics with respondents recruited from a study exploring the intersection of housing and health using virtual focus groups with Métis people between August 2022 and February 2023.

| **Please select the statement that best describes your use of the Zoom Video Conference Tool:**  This was my first time using Zoom  I have used Zoom before, but I do not use Zoom regularly  I use Zoom regularly for work, school, personal use, etc. |
| --- |
| **I am confident in my ability to use the Zoom Video Conference tool:**  Strongly agree  Somewhat agree  Neither agree nor disagree  Somewhat disagree  Strongly disagree |
| **I was satisfied with participating in a focus group online:**  Strongly agree  Somewhat agree  Neither agree nor disagree  Somewhat disagree  Strongly disagree |
| **I would have preferred to participate in a focus group in person:**  Strongly agree  Somewhat agree  Neither agree nor disagree  Somewhat disagree  Strongly disagree |
| **In the future, I would like to participate in other online focus groups:**  Strongly agree  Somewhat agree  Neither agree nor disagree  Somewhat disagree  Strongly disagree |
| **It is more feasible for me to participate in an online focus group than an in-person focus group:**  Strongly agree  Somewhat agree  Neither agree nor disagree  Somewhat disagree  Strongly disagree |
| **I believe it is culturally appropriate to use virtual focus groups for research involving Citizens of the Métis Nation of Ontario:**  Strongly agree  Somewhat agree  Neither agree nor disagree  Somewhat disagree  Strongly disagree |
| **I felt that my information will be kept confidential by the research team:**  Strongly agree  Somewhat agree  Neither agree nor disagree  Somewhat disagree  Strongly disagree |
| **I believe the other participants were in a private space:**  Strongly agree  Somewhat agree  Neither agree nor disagree  Somewhat disagree  Strongly disagree |
| **I believe my information will be kept confidential by the other people who participated in the focus group:**  Strongly agree  Somewhat agree  Neither agree nor disagree  Somewhat disagree  Strongly disagree |
| **I was satisfied with the compensation I received for participating in the study:**  Strongly agree  Somewhat agree  Neither agree nor disagree  Somewhat disagree  Strongly disagree |
| **Please select the statement that best describes the quality of your Zoom Video Conference tool experience:**  **I was able to see the other participants during the focus group discussion (Video quality):**  All of the time  Most of the time  Some of the time  Rarely  None of the time  **I was able to hear the other participants during the focus group discussion (audio quality):**  All of the time  Most of the time  Some of the time  Rarely  None of the time  **I was able to hear and see the other participants without delays in the live feed (lag in audio and/or video):**  All of the time  Most of the time  Some of the time  Rarely  None of the time  **I feel that I am able to connect with other participants in a meaningful way during the focus group discussion:**  All of the time  Most of the time  Some of the time  Rarely  None of the time |
| **Is there anything else you would like to tell us about conducting focus groups using Zoom?**  Open text-box answer |
